# Detection of Persistent Viruses by High-Throughput Sequencing in Tomato and Pepper from Panama: Phylogenetic and Evolutionary Studies

**DOI:** 10.3390/plants10112295

**Published:** 2021-10-26

**Authors:** Luis Galipienso, Laura Elvira-González, Leonardo Velasco, José Ángel Herrera-Vásquez, Luis Rubio

**Affiliations:** 1 Plant Protection and Biotechnology Center of the Valencian Institute of Agricultural Research, 46113 Moncada, Valencia, Spain; lrubio@ivia.es; 2 Subtropical and Mediterranean Horticulture Institute (LaMayora), 29010 Algarrobo-Costa, Málaga, Spain; Laura.e.g@csic.es; 3 Churriana Center of Andalusian Institute of Agricultural Research, 29140 Churriana, Málaga, Spain; Leonardo.velasco@juntadeandalucia.es; 4 Divisa Center of the Panamanian Agricultural and Innovation Institute, Divisa 0619, Herrera, Panama; jose.herrera@idiap.gob.pa

**Keywords:** high throughput sequencing, STV, BPEV, pepper, tomato, nucleotide diversity, recombination

## Abstract

High-throughput sequencing from symptomatic tomato and pepper plants collected in Panama rendered the complete genome of the southern tomato virus (isolate STV_Panama) and bell pepper endornavirus (isolate BPEV_Panama), and almost-complete genomes of three other BPEV isolates. Tomato chlorosis virus, tomato mosaic virus, and impatiens necrotic spot virus were also detected. Analysis of the complete genome of STV and BPEV worldwide isolates revealed nucleotide diversities of 0.004246 and 0.070523, respectively. Bayesian phylogenetic analysis showed two main groups for each virus (I and II), and several subgroups for BPEV (IA, IB, IC, IIA and IIB). Isolate STV_Panama clustered with NC_12-03-08 from USA and Tom3-T from France (99.97% nucleotide identity) in Group I and BPEV_Panama was close to the Canadian isolate BPEV_Ontario (99.66% nucleotide identity) in Subgroup IB. No correlation was observed between geographic and genetic distances for both viruses. Panamanian BPEV isolates were divergent, belonging to Groups I and II (nucleotide identities > 87.33%). Evolutionary analysis showed purifying selection in all encoding regions of both viruses, being stronger in the overlapping region of both STV genes. Finally, recombination was detected in BPEV but not in STV. This is the first report of STV and BPEV in Panama.

## 1. Introduction

Some viruses exhibit persistent lifestyle in their host plants through vertical seed transmission. Persistent viruses consist, in general, of double stranded RNA (dsRNA) genomes that replicate in the cell cytoplasm, and some of them are unable to form viral particles [1]. Despite persistent plant viruses being widespread and infecting many plant species, they are poorly studied, since historically many were considered non-pathogenic viruses. Some of them, such as the white clover cryptic virus 1 (WCCV-1) and curvularia thermal tolerance virus (CThTV), establish symbiotic relationships with their hosts [2,3]. Nevertheless, recent studies have shown that southern tomato virus (STV), a persistent virus infecting tomato (*Solanum lycopersicum*), interacts with cucumber mosaic virus (CMV) and pepino mosaic virus (PepMV) in mixed infections by increasing the pathogenic effect of these viruses [4]. Besides this, the presence of STV-induced changes with the accumulation of some regulatory micro-RNAs (miRNAs) that target plant genes involved in essential plant processes such as plant development and responses to biotic or abiotic stress [4,5]. Interaction of persistent and pathogenic plant viruses is an important issue that needs to be further studied, since mixed infections are very frequent in natural conditions.

Southern tomato virus (STV) belongs to the genus *Amalgavirus* (family *Amalgaviridae*) and infects tomato (*Solanum lycopersicum*). STV consists of a dsRNA genome of 3.5 kb encoding for two proteins: the putative coat protein (Cp) named p42, and the RNA-dependent RNA-polymerase (RdRp), a fusion protein that is expressed by a +1 ribosomal frameshift [6]. STV is spread worldwide, and the only known way of virus transmission is vertically via seeds, with rates of up to 80%. Recent surveys in tomato commercial crops and nurseries from Spain showed a high incidence of STV [7,8]. Bell pepper endornavirus (BPEV) is another persistent virus belonging to the genus *Alphaendornavirus* (family *Endornaviridae*), which infects pepper (*Capsicum* sp.) [9]. BPEV has a dsRNA genome of approximately 14.7 kb containing a single open reading frame (ORF), which is translated into a large polyprotein of 4815–4884 aa. This polyprotein contains several conserved functional domains, such as putative viral methyltransferase (MTR), helicase 1 (Hel-1), UDP-glycosyltransferase (UDG) and RNA-dependent RNA polymerase (RdRp). It is hypothesized that this polyprotein is cleaved into functional domains, but the viral protease has not been identified. BPEV is widespread, and like STV, the only known way of virus transmission is vertically via seeds, with an efficiency of up to 100% [10].

Metagenomic studies by high-throughput sequencing (HTS) have revealed that plants are frequently infected by persistent viruses, allowing the increase of nucleotide datasets of these viruses over recent years [11,12]. In this work, we have detected STV and BPEV by HTS of small RNAs (sRNAs) from symptomatic tomato and pepper samples, respectively, collected in Panama. The presence of both viruses was confirmed by real-time PCR (RT-qPCR) using specific primer sets. Complete genome sequences of one Panamanian isolate each of STV and BPEV, and partial BPEV sequences of additional isolates were obtained. HTS also revealed the presence of some pathogenic viruses such as tomato mosaic virus (ToMV) and tomato chlorosis virus (ToCV) in tomato or impatiens necrotic spot virus (INSV) in pepper, which induce similar symptoms to those observed in the collected plants.

Tomato and pepper are important horticultural crops worldwide, with a production of around 180 and 38 million tons (T) in 2019, respectively [13]. In Panama, tomato production has been increasing over the recent years, due to the interest of the food processing industry, to reach around 15,000 T [14]. Pepper is another important horticultural crop in the region, with a production of around 7000 T [14], being used mainly as a condiment or fresh food.

To our knowledge, this is the first report of STV and BPEV in Panama. We also analyzed the genetic diversity and evolutionary parameters of STV and BPEV, that will contribute to the better understanding of the genetic complexity of these viruses on a global scale. In addition, analyses of the genetic diversity and evolution of STV and BPEV are crucial to understanding their epidemiology, and to developing accurate detection methods [15].

## 2. Results and Discussion

### 2.1. High-Throughput Sequencing of Small RNAs

Virus detection by HTS of sRNAs is a useful technique for the diagnosis and discovery of plant viruses [16,17]. The plant material used in this work and the results obtained by HTS (includinf the VirusDetect output) of sRNAs using the Illumina NextSeq550 platform are summarized in Table 1 and in Supplementary Table S1, respectively. For tomato (sample 1), leaf tissue of three symptomatic plants were collected in a plot located at Palma Real (Chiriquí province) and processed in a single pool for sequencing. Blastn and Blastx analyses of the de novo assembled contigs indicated the presence of persistent STV, which was confirmed by RT-qPCR. Sanger sequencing of the amplification products showed 100% homology with the STV nucleotide sequence obtained by HTS. In addition to STV, Blast analysis revealed the presence of the pathogenic broad bean stain virus (BBSV), tomato chlorosis virus (ToCV) and tomato mosaic virus (ToMV) in that sample. For BBSV, only a short contig (60 nt) showing 91% of nucleotide identity with the BBSV available sequence of 276 nt (KJ746622) was observed. Further analysis showed that no reads matched to any BBSV nucleotide sequence available in GenBank. Finally, no amplification products were obtained after using the same total RNAs extracts used for HTS in a RT-PCR assay performed with a pair of specific primers designed on that nucleotide sequence (data not shown). Thus, this BBSV contig could be considered as an assembly artifact. ToCV and/or ToMV could be responsible for the stunting, chlorosis and brown necrosis symptoms that were observed in the collected tomato plants since Blastx analysis did not show the presence of any potential new viruses in the tomato sample.

In pepper, leaf tissues of three symptomatic plants were collected from each plot at El Ejido (Los Santos province), Tierra Blanca (Los Santos province) and San Ramón (Chiriquí province), consisting of samples 2, 3 and 4, respectively. In each plot, tissues of three individual plants were processed in a single pool for HTS. Blastn and Blastx analyses of the de novo unique assembled contigs revealed only the presence of BPEV in samples 2 and 3, whereas in sample 4, INSV was detected in addition to BPEV (Supplementary Table S1). The presence of BPEV was confirmed in all samples by RT-qPCR, and Sanger sequencing of amplification products showed 100% of homologies with the BPEV nucleotide sequence obtained by HTS.

Plants of sample 2 showed strong leaf deformation and curling; plants of sample 3 showed leaf curling, mosaic and brown necrosis, whereas plants of sample 4 showed leaf mosaic and black spotted necrosis. As BPEV has never been associated with plant symptoms until now, symptoms in sample 2 and 3 could have been induced by potential unknown viruses with low sequence identity with records in virus databases, since no significant matches were obtained in the Blatx analysis (Supplementary Table S1), or, by some other unknown pathogenic organisms. Alternatively, an abiotic origin as consequence of phytotoxicity or nutritional deficiencies cannot be discarded. Plants of sample 4 showed black spotted necrosis on the leaves, identical to those induced by INSV.

### 2.2. Genetic Diversity

Complete genome sequences of two isolates—one of STV (STV_Panama, MT051992) and another of BPEV (BPEV_Panama, MZ127290)—from Panama were obtained from sample 1 (Palma Real) and sample 4 (San Ramón), respectively. STV_Panama and BPEV_Panama sequences were aligned to the complete genome sequences of respective viruses available in GenBank (Supplementary Tables S2 and S3, respectively). The complete genome sequence of STV_Panama was obtained from a single contig of 3438 nucleotides (nts) that showed a nucleotide identity higher than 98.0% with all the STV sequences available in GenBank. Pairwise nucleotide identity analysis (percentage of positions with identical nucleotides in pairwise comparisons) with STV complete genome sequences (Supplementary Table S4) showed that the isolates most closely related to STV_Panama were NC_12-03-08 from the USA and Tom3-T from France (99.97% for both isolates), whereas the most distant isolates were CH_bpo161 and CH_bpo163 from Switzerland (98.43 and 98.46%, respectively). The nucleotide diversity (mean of nucleotide distance among virus isolates) of STV was low (0.004246 ± 0.000536), with the 3′and 5′ non-coding regions (UTRs) being the most conserved (0.000860 ± 0.000083 and 0.002805 ± 0.001008, respectively). Regarding the two protein-encoding regions, the nucleotide diversities of p42 and RdRp were 0.0048365 ± 0.001071 and 0.004487 ± 0.000470, respectively. Low nucleotide diversity was also obtained in a previous report for the putative capsid-encoding regions of STV and in other amalgaviruses, such as blueberry latent virus (BBLV) [18].

The complete genome sequence of BPEV_Panama was also obtained from a single contig of 14,715 nts, and showed nucleotide identities ranging from 99.76 to 86.27% with the BPEV sequences available in GenBank. Pairwise complete genome sequence analysis of BPEV (Supplementary Table S5) showed that the isolate BPEV_Panama was closely related to isolate BPEV_Ontario (99.66%) from Canada and distantly related to isolates BPEV_TW from India and BPEV_XJ from China (86.28 and 86.27%, respectively). The nucleotide diversity of BPEV was low (0.070523 ± 0.001368), but higher than that of STV, with the 5´UTR being the most conserved region in BPEV (0.02667 ± 0.024112), while the polyprotein and the 3´UTRs represented less conserved regions (0.070501 ± 0.001378 and 0.160635 ± 0.039341, respectively).

In addition to the complete genome sequence of BPEV obtained in sample 4, partial BPEV sequences were obtained by HTS from samples 2 and 3. Sample 2 (El Ejido), provided 57 contigs covering 87.9% of the full genome sequence of the BPEV-El Ejido isolate. Sample 3 (Tierra Blanca) provided 136 contigs and was the host of two divergent BPEV isolates, named BPEV_Tierra Blanca 1 and 2, covering 94.2 and 88.1% of the BPEV genome, respectively, and showing a nucleotide identity of 87.80% when compared between them. Non-overlapping contigs of each BPEV isolate were concatenated and individually aligned with the complete genomic sequences of BPEV used in this work. Analysis of pairwise nucleotide identities (Supplementary Table S6) showed that isolate BPEV_El Ejido was closely related to the North American isolate BPEV-YW (97.77%) and distantly related with the Chinese isolate BPEV_lj A (87.18%). With respect to the isolates identified in sample 3, BPEV_Tierra Blanca 1 was found to be closely related to the Dominican isolate BPEV_DR (99.20%), and distantly related to the Indian isolate BPEV_TW (87.25%). In contrast, BPEV_Tierra Blanca 2 was closely related to the North American isolate BPEV-YW (97.80%) and distantly related to the Chinese isolate BPEV_lj (87.47%). Among BPEV Panamanian isolates, BPEV_Panama was closely related to BPEV_Tierra Blanca 1 (99.10%) and distantly related to both isolates BPEV_Tierra Blanca 2 and BPEV_El Ejido (87.66 and 87.33%, respectively) that, conversely, were closely related to each other (98.89%).

### 2.3. Phylogenetic Analysis

**Figure 1:**
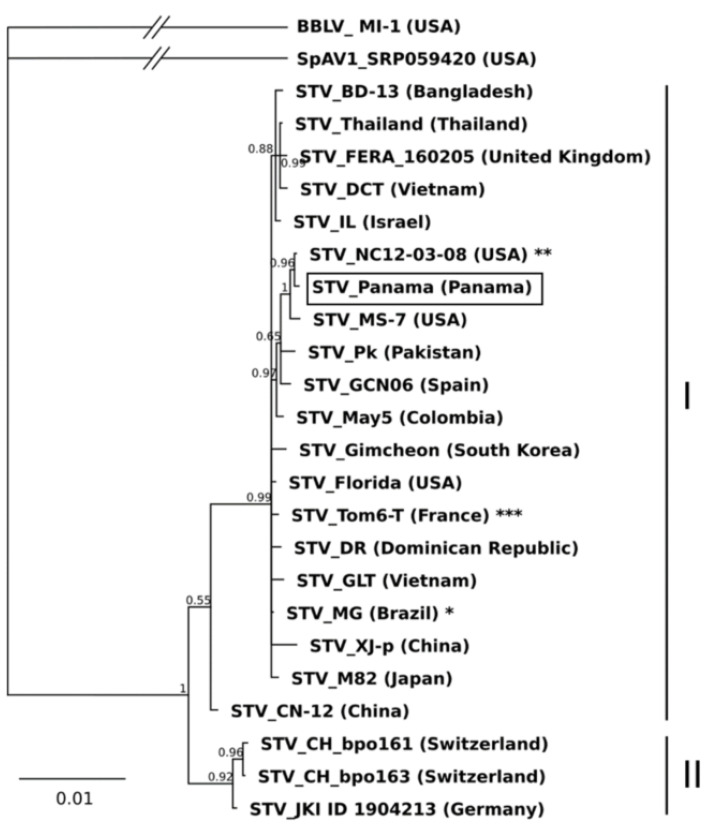
Bayesian phylogenetic tree generated from the complete genome sequences of southern tomato virus (STV) showing two groups or clades: I and II. Posterior probabilities higher than 0.65 are shown in nodes. Asterisks indicate several STV isolates with identical sequences: (*) STV_Canada (Canada), STV_DTT (Vietnam), STV_DCT (Vietnam) and STV_Mexico-1 (Mexico); (**) STV_Tom3-T (France); (***) STV_Tom5-N, STV_Tom5-T and STV_Tom1-T from France. The STV isolate obtained in this study is highlighted with a rectangle. Blueberry latent amalgavirus (isolate BBLV_M1-1 from USA, HM029246) and Spinach amalgavirus 1 (isolate SpAV1_SRP059420 from USA, KY695011) were used as the outgroup.

**Figure 2:**
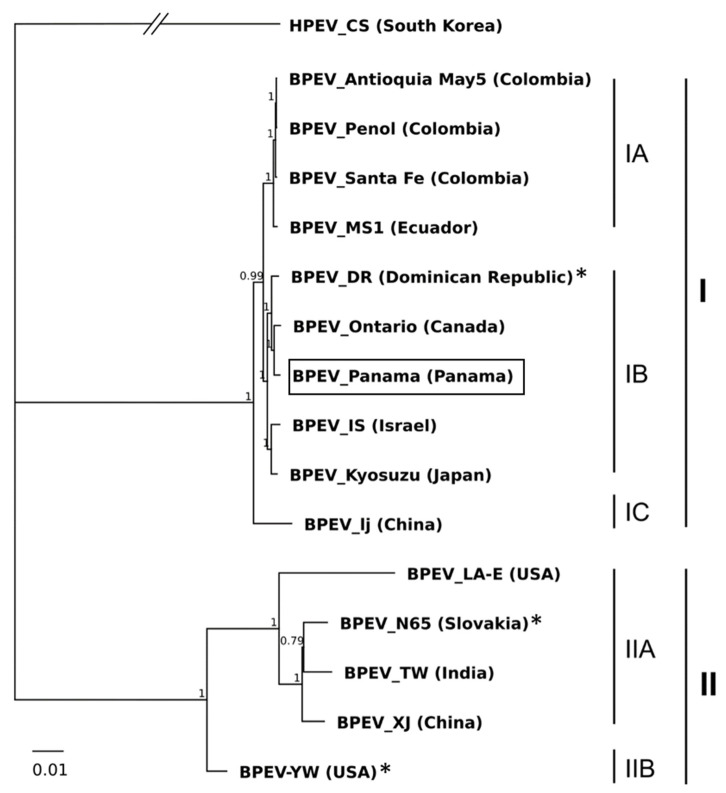
Bayesian phylogenetic tree generated from the complete genome sequences of bell pepper endornavirus (BPEV) showing two main groups or clades I and II and subgroups IA, IB, IC, IIA and IIB. Posterior probabilities higher than 0.65 are shown in nodes. The BPEV isolate obtained in this study is highlighted with a rectangle and recombinant isolates (Section 2.4) with an asterisk (*). Hot pepper endornavirus (isolate HPEV_CS from South Korea, NC_027920) was used as outgroup.

Phylogenetic analysis of the STV and BPEV complete genome sequences showed that the best fitting nucleotide substitution model was the Tamura-Nei [19] for both viruses. The Bayesian phylogenetic trees showed two clades or groups for both STV and BPEV (I and II; Figure 1 and Figure 2, respectively). In the phylogenetic analysis, STV_Panama was ascribed to Group I, which also contained STV isolates from Spain, France, Mexico, UK, Dominican Republic, USA, Canada, Brazil, South Korea, Colombia, Israel, Vietnam, Thailand, Bangladesh, Japan, Pakistan and China. Group II contained only isolates from Switzerland and Germany. The nucleotide diversity within groups I and II were 0.002131 ± 0.000316 and 0.000984 ± 0.000411, respectively, and the nucleotide distance between both groups was 0.013833 ± 0.002152. The tree topology obtained with Bayesian analysis from the complete genome sequences of STV was similar to that obtained in a previous study using the maximum likelihood (ML) method from the putative capsid p42 region [18]. However, one difference was found: isolate STV_CN12 from China clustered in Group B (equivalent to II) with ML analysis and in Group I with the Bayesian analysis. In either of the two studies, no correlation was found between the geographic and nucleotide distances, and some isolates from different countries showed identical nucleotide sequences, such as STV_Mexico-1 from Mexico, STV_DTT and STV_DDT from Vietnam, STV_Canada from Canada and STV_MG from Brazil; Tom3-T from France and NC12-03-08 from the USA; or Tom5-N, Tom5-T, Tom1-T and Tom6-T from France.

In the phylogenetic analysis of BPEV isolates (Figure 2), BPEV_Panama was ascribed to Group I, which also contained isolates from Canada, Ecuador, Dominican Republic, Colombia, Israel, Japan, and China. Group II contained isolates from the USA, Slovakia, India and China. The nucleotide diversity within groups I and II were 0.0008719 ± 0.000365 and 0.036405 ± 0.001232, respectively, and the nucleotide distance between both groups was 0.132971 ± 0.002528.

Group I was split into three subgroups: IA, containing isolates from Colombia and Ecuador; IB, containing isolates from the Dominican Republic, Panama, Canada, Israel and Japan; and IC, which included the isolate BPEV-lj from China. Group II was also separated in subgroups: IIA, with isolates from India, Slovakia and USA, and IIB with the isolate BPEV-YW from USA. The nucleotide diversity within subgroups IA, IB and IIA, were 0.001125± 0.000193, 0.005153 ± 0.000392, and 0.031048 ± 0.001053, respectively. Regarding the partial sequences of Panamanian isolates, BPEV_El Ejido and BPEV_Tierra Blanca 2, were ascribed to Subgroup IIB, close to isolate BPEV-YW from the USA—whereas BPEV_Tierra Blanca 1 was placed in Subgroup IB, close to the isolate BPEV_DR from the Dominican Republic and BPEV-Panama. The presence of BPEV isolates clustering in different phylogenetic groups indicates that the virus population in Panama is not uniform.

The results of STV and BPEV phylogenetic analysis reported here are in accordance with previous studies, although for BPEV, the main groups were not previously divided into subgroups [1,18,20]. For both STV and BPEV, a geographical structuring of virus populations was not found; STV isolates from different countries had identical nucleotide sequences while, on the other hand, BPEV isolates of the same country were clustered into different phylogenetic groups. These results could be explained by the international trade of infected tomato and pepper seeds. BPEV isolates from the same country clustering in different phylogenetic groups were reported previously in Slovakia, determining that the virus population was not genetically uniform [20]. Moreover, in our work, isolates BPEV_Tierra Blanca 1 and 2 were detected in the same pepper sample. As samples were a mix of three individual plants, it is not possible to ensure if isolates were infecting the same or different plants. Nevertheless, the three individual plants were collected in the same plot, showed the same symptoms and corresponded to the same local variety (IDIAP149). BPEV phylogeny has been correlated with host pepper genotypes as a consequence of breeding activities and the long co-existence between the virus and its host [1,10,21]. Here, we report the presence of two genetically different BPEV isolates in the same pepper local variety, which is locally cultivated, and its seeds obtained by the farmers themselves. Cross-pollination of plants containing different BPEV isolates could contribute to the generation of mixed infections that facilitate the recombination process, contributing to virus evolution.

### 2.4. Natural Selection and Recombination Analysis

The role of natural selection and recombination in STV and BPEV evolution was investigated. Natural selection was analyzed by comparing the rates of non-synonymous substitutions (dN) and synonymous substitutions (dS) in both the whole encoding regions and individually at each codon across the virus genomes by using the Pamilo–Bianchi-Li and FUBAR methods, respectively [22,23,24]. For STV, values of dN and dS for p42 were 0.001118 ± 0.000564 and 0.011732 ± 0.002966, respectively, and for RdRp were 0.001791 ± 0.000375 and 0.009478 ± 0.001353, respectively. The value of the dN/dS for p42 and RdRp were 0.095294 and 0.188963, respectively—indicating a purifying/negative selection for amino acid changes in both proteins, being stronger for p42 than for RdRp. The value of dN/dS for p42 obtained in this work is slightly lower than that reported in a previous work using different STV isolates [18]. In addition, dN and dS of RdRp were calculated in the p421-RdRp overlapping and non-overlapping regions. For the overlapping region (from the protein start to the frameshift site), the values of dN and dS were 0.001168 ± 0.000437 and 0.009307 ± 0.002103, respectively, and dN/dS was 0.125496. For the non-overlapping regions, the values of dN and dS were 0.002091 ± 0.000538 and 0.010017 ± 0.001661, respectively, and dN/dS was 0.208745. As expected, the purifying selection for RdRP was stronger in the p42-RdRp overlapping region, since natural selection acts in two proteins. Additionally, analysis of the natural selection pressure in individual codons showed purifying selection at 13 sites in p42 (31, 36, 40, 62, 65, 74, 83, 138, 157, 185, 197, 218, 239) and 10 sites in RdRp (31, 36, 62, 83, 157, 185, 407, 485, 566, 665), whereas one site (464) with positive/diversifying selection was detected in the RdRp (posterior probability of 0.9). For BPEV, values of dN and dS for the polyprotein were 0.027110 ± 0.001179 and 0.237821 ± 0.006365, respectively, and dN/dS was 0.113993—suggesting that purifying selection was responsible for amino acid changes. Analysis of natural selection in individual codons across the BPEV polyprotein showed 1877 sites under purifying selection and five sites (969, 963, 3685, 3702, 2798) with positive/diversifying selection (posterior probability of 0.9).

Overall, values of dN/dS for STV and BPEV polyprotein were low but in the range of the majority of plant viruses so far described [25]. Purifying selection in all the encoding regions of STV and BPEV suggest that these proteins are under functional or structural constraints. For STV, most of these sites in the RdRp were found in the p42 overlapping region (31, 36, 62, 83, 157, 185), despite this region (253 residues) being shorter than the non-overlapping region (809 residues)—whereas the only diversifying position of RdRp was in the p42 non-overlapping region. The biological role of p42 remains unclear; it has been suggested that it is the STV capsid protein, but virions have been never observed and no homologies with other viral capsid proteins have been found [6]. Moreover, recent studies showed that p42 has no RNA silencing suppressor activity [4]. Differences in the purifying selection pressure in both p42 overlapping and non–overlapping regions found in RdRp, with stronger pressure in the first one, reinforce the hypothesis of the in vivo expression of p42—although its biological function remains unknown.

No recombination was detected among the 31 complete genome sequences of STV (Supplementary Table S2) after analysis with the RDP5 program. Recombination in STV might be not detected due to the low nucleotide distances between STV sequences. On the other hand, six recombination events (Table 2) were detected with this same software when 15 complete nucleotide sequences of BPEV (Supplementary Table S3) were studied: four in isolate BPEV_YW, one in BPEV_DR and one in BPEV_N65. No recombination events were detected in isolate BPEV_Panama. Three out of six events, all of them found in isolate BPEV_YW, were detected in seven out of the ten recombination methods implemented in RDP5 program: In event nº1 (recombination sites 4860-5570), the major parent was isolate BPEV_N65 (Slovakia, Subgroup B1) and the minor parent was isolate BPEV_MS1 (Ecuador, Subgroup A1); in event nº 2 (recombination sites 6350-7162) the major parent was isolate BPEV_XJ (China, Subgroup B1) and the minor parent was BPEV_MS1 (Ecuador, Subgroup A1); in event nº 3 (recombination sites 24-286), the major parent was isolate BPEV_N65 (Slovakia, Subgroup B1) and the minor parent was isolate BPEV_Ontario (Canada, Subgroup A2). In a previous study, only two recombination events in similar positions to events nº1 and 2 reported here were found in isolate BPEV_YW [18]. Recombination events showed the same major parent in both studies but different minor parents. For BPEV, the presence of two main genetically distant groups allowed the identification of recombination events among the isolates of these groups. Absence of recombination would be expected for persistent viruses, since vertical transmission would prevent the coexistence of different virus variants in the same cell. However, some recombination events might occur by fusion of gametic cells infected with different virus variants.

## 3. Materials and Methods

### 3.1. Plant Material, Sample Preparation and High-Throughput Sequencing

Leaf tissues from tomato and pepper plants showing typical symptoms of viral infection were collected in four plots in different geographical areasof Panama in the dry season of 2018 (Table 1). For each sample, one corresponding to tomato (sample 1) and three to pepper (samples 2, 3 and 4) leaf tissues of three individual plants of the same plot showing identical symptoms were collected in a single pool, desiccated in silica gel and stored at room temperature until processing. Total RNA was extracted by using the Spectrum Plant Total RNA Kit (Sigma-Aldrich, San Luis, MO, USA) following the manufacturer´s instructions, and used for HTS of small RNAs. RNA concentration and purity were determined by using the Qubit^®^ RNA assay Kit in the Qubit^®^ 3.0 Fluorometer (ThermoFisher, Waltham, MA, USA) and the NanoPhotometer^®^ spectrophotometer (Implen, Westlake Village, CA, USA), respectively. RNA integrity was determined in the Agilent Bioanalyzer 2100 system with the RNA Nano 6000 assay kit (Agilent Technologies, Santa Clara, CA, USA). cDNA was obtained from 1 µg of total RNA of each sample by using the NEBNext^®^ Multiplex Small RNA library Prep Set for Illumina^®^ (Sigma-Aldrich, San Luis, MO, USA) and sequenced by using the Illumina NextSeq550 platform (Illumina, San Diego, CA, USA). cDNA libraries were uploaded to the NCBI platform and published under the Bioprojects PRJNA720388 and PRJNA734294. Reads were cleaned by trimming the sequencing adapters and low-quality reads were filtered by using SeqTrimNext V2.0.67 software in January 2020 (https://github.com/dariogf/SeqtrimNext)—a next-generation sequencing-evolved version of SeqTrim—applying the standard parameters for Illumina short reads [26]. High-quality trimmed reads were further analysed for virus identification in January, 2020 with the VirusDetect V 1.7 [27] by using the custom virus reference database (http://bioinfo.bti.cornell.edu/ftp/program/VirusDetect/virus_database/v239/, accessed on 1 August 2021). Firstly, reads were aligned with the virus nucleotide database by using the Burrows–Wheler Aligner program [28], and then, aligning reads were de novo assembled using Velvet V.1.2.09 software [29] with automated parameter optimization. To avoid the loss of nucleotide sequences of novel viruses, reads not aligning with viral sequences were recovered and de novo assembled as described above. De novo generated contigs were compared to the nucleotide and protein database using Blastn and Blastx, respectively, with an e-value of 10 × 10^−5^ and a minimum identity percentage of 25%.

### 3.2. Virus Detection by RT-qPCR Assay and Sanger Sequencing

STV detection was performed by RT-qPCR with primers and a TaqMan probe set previously designed in the CP (1189–1257 nts) region [30], whereas for BPEV detection, a set of primers and a GMP probe were designed by using the IDT online Realtime PCR Tool (https://eu.idtdna.com/scitools/Applications/RealTimePCR) in August 2021, on the basis of a conserved region (11875-1293) obtained by aligning the nucleotide sequences of the two most divergent BPEV isolates (BPEV_Panama and BPEV_El Ejido; Supplementary Table S7). STV and BPEV probes were tagged with the fluorescent dye 6FAM and the quencher TAMRA at the 5′ and 3′ terminal nucleotide sequences, respectively. RT-qPCR was performed with the One step PrimeScript RT-PCR Kit (TaKaRa, Shiga, Japan) in a Light-Cyler 480 (Roche, Basilea, Switzerland) following the manufacturer’s instructions, with some modifications. The total RNA extracts (50 ng) were denaturalized in the presence of 0.2 µM of both forward and reverse primers 95 °C for 5 min. Subsequently, a mix containing 10 µL of one-step RT-PCR buffer III, 2 U Ex Taq HS, 0.4 µL PrimeScript RT Enzyme Mix II, and 0.2 µM specific TaqMan probe was added to a final volume of 20 μL. The thermal cycling conditions were: reverse transcription at 42 °C for 15 min, incubation at 94 °C for 10 s, and 40 cycles of DNA amplification at 94 °C for 5 s and 60 °C for 20 s. The total RNA extracts of non-infected tomato and pepper plants were used as negative RT-qPCR controls. To confirm the STV and BPEV identity of amplicons, RT-qPCR products were purified using a QIAquick PCR Purification Kit (Qiagen, CA, USA) and sequenced by sanger using a Big Dye Terminator V. 3.0 Cycle Sequencing kit in an ABI 3130 XL capillary sequencer (Applied Biosystems, CA, USA).

### 3.3. Sequence Analysis

In addition to the complete genome sequences of the STV and BPEV isolates from Panama determined here, genomes of both viruses from different countries not containing nucleotide indeterminations were retrieved from GenBank (Supplementary Tables S2 and S3, respectively). Viral nucleotide sequences were aligned at the amino acid level using the program CLUSTAL W 2.0 [31], and the nucleotide substitution models that best fitted the sequence data were determined to infer their phylogenetic relationships. The phylogenetic trees were generated using the Mr. Bayes plugin [32] available in Geneious v.2019 (Biomatters, Auckland, New Zeland). The role of natural selection at the molecular level was evaluated by comparing the rate of nonsynonymous substitutions per nonsynonymous site (dN) and the rate of synonymous substitutions per synonymous site (dS), according to the Pamilo–Bianchi–Li method [22]. All these analyses were performed with MEGA X [33]. Selection across the genomic coding regions was studied by estimation of the rates of dN and dS at each codon using the FUBAR method [23,24], which is implemented in the Datamonkey Server (https://www.datamonkey.org/) [34] in May 2021, and uses a Bayesian approach. Recombination among virus isolates was assessed by the program RDP v.4.97 [35].

## Figures and Tables

**Table 1:** Samples of tomato (*Solanum lycopersicum*) and pepper (*Capsicum annuum*) collected from different areas of Panama and used for high-throughput sequencing (HTS).

Sample	Locality	Coordinates	Host	Symptoms
1	Palma Real (Potrerillo, Dolega, Chiriquí)	8°39′49′′ N 82°31′12′′ W	*S. lycopersicum*	Plant stunting, chlorosis and brown necrosis
2	El Ejido (El Ejido, Los Santos)	7°54′18′′ N 80°22′03′′ W	*C. annuum*	Leaf deformation and curling
3	Tierra Blanca (El Espinal, Guararé, Los Santos)	7°50′11′′ N 80°20′23′′ W	*C. annuum*	Leaf curling, mosaic and brown necrosis
4	San Ramón (Los Naranjos, Boquete, Chiriquí)	8°48′47′′ N 82°27′42′′ W	*C. annuum*	Leaf mosaic and black spotted necrosis

**Table 2:** Recombination results obtained by using the RDP5 program from the complete nucleotide sequences of bell pepper endornavirus (BPEV) isolates.

Event	Position	Isolate	Major Parent	Minor Parent	Method *
1	4860–5570	BPEV-YW (JN019858)	BPEV_N65 (MN580384)	BPEV_MS1 (MN175323)	R, G, B, M, C, S, 3S
2	6350–7162	BPEV-YW (JN019858)	BPEV_XJ (MH182675)	BPEV_MS1 (MN175323)	R, G, B, M, C, S, 3S
3	24–286	BPEV-YW (JN019858)	BPEV_N65 (MN580384)	BPEV_Ontario (KT149366)	R, G, B, M, C. 3S
4	14591–14610	BPEV-YW (JN019858)	BPEV_TW (KU923756)	BPEV_Ontario (KT149366)	G, B, M, S, 3S
5	14724–14728	BPEV_DR (KX525267)	BPEV_Ontario (KT149366)	Unknown	G, B, M, 3S
6	14662–14756	BPEV_N65 (MN580384)	BPEV_XJ (MH182675)	BPEV_lj (KF709944)	G, B, 3S

* Recombination detection methods: R: RDP, G: GENECONV, B: BootScan, M: MaxChi, C: Chimera, S: SiScan, 3S: 3Seq.

## Data Availability

The data presented in this study are available on request from the corresponding author.

## Supplementary Materials

The following are available online at https://www.mdpi.com/article/10.3390/plants10112295/s1. Table S1: Results of high-throughput sequencing (HTS) of small RNAs of tomato (*Solanum lycopersicum*) and pepper (*Capsicum annuum*) samples; Table S2: List of southern tomato virus (STV) isolates from tomato (*Solanum lycopersicum*) used in this study. The complete genome sequence of STV_Panama was determined in this work and the rest retrieved from GenBank; Table S3: List of isolates of bell pepper endornavirus (BPEV) from pepper (*Capsicum annumm* and *C. frutescens*) used in this study. The complete genome sequence of isolate BPEV_Panama and partial (concatenated) sequences of isolates, BPEV_El Ejido, BPEV_Tierra Blanca 1 and 2 were determined in this work and the rest were retrieved from GenBank; Table S4: Pairwise nucleotide identities among the complete genome sequences of different southern tomato virus (STV) isolates; Table S5: Pairwise nucleotide identities among the complete genome sequences of different bell pepper endornavirus (BPEV) isolates; Table S6: Pairwise nucleotide identities among the partial nucleotide (concatenated) and the complete genome sequences of different bell pepper endornavirus (BPEV) isolates; Table S7: Primers and probes used for detection of southern tomato virus (STV) and bell pepper endornavirus (BPEV) by RT-qPCR.
