# Supplementary material for: Detection of Persistent Viruses by High-Throughput Sequencing in Tomato and Pepper from Panama: Phylogenetic and Evolutionary Studies
Source: Plants (Basel). 2021 Oct 26;10(11):2295. doi: 10.3390/plants10112295 (PMC8620285; doi:10.3390/plants10112295)
Supplement: Supplementary file 1 [file plants-10-02295-s001.zip › Suplementary Table S2 (Galipienso et al., ).pdf]

Isolate	Country	Accession n.	Tomato variety	Collection year
STV_Panama	Panama	MT051992	Local	2019
STV_JKI ID 1904213	Germany	MK948544	Unknown	2018
STV_Florida	USA	KX949574	Sweet hearts	2013
STV_NC12-03-08	USA	KT852573	Unknown	2012
STV_MS-7	USA	EU413670	Unknown	2005
STV_Thailand	Thailand	LC487710	Unknown	2019
STV_M82	Japan	LC429302	M82	2018
STV_MG	Brazil	MG808383	Dominator	2013
STV_IL	Israel	MH356747	Unknown	2017
STV_GCN06	Spain	KJ174690	Mariana	2006
STV_BD-13	Bangladesh	KT634055	Unknown	2013
STV_Mexico-1	Mexico	EF442780	Unknown	2005
STV_DDT	Vietnam	MW012413	Unknown	2017
STV_DCT	Vietnam	MW012412	Unknown	2017
STV_DTT	Vietnam	MW012411	Unknown	2017
STV_GLT	Vietnam	MW012410	Unknown	2017
STV_Pk	Pakistan	MT066231	Rio Grande	2019
STV_May5	Colombia	MN095716	Unknown	2018
STV_Tom6-T	France	MN216389	Unknown	2018
STV_Tom5-N	France	MN216388	Unknown	2018
STV_Tom5-T	France	MN216387	Unknown	2018
STV_Tom3-T	France	MN216385	Unknown	2018
STV_Tom1-T	France	MN216384	Unknown	2018
STV_Canada	Canada	MK610257	Premio	2019
STV_CH_bpo163	Switzerland	MF422618	Merlice	2014
STV_CH_bpo161	Switzerland	MF422617	Merlice	2014
STV_FERA_160205	UK	KY810783	Unknown	2016

STV_DR	Dominican Republic	KX525266	Unknow	2013
STV_XJ-p	China	KY228384	Unknow	2015
STV_CN-12	China	KT438549	Unknow	2012
STV_Gimcheon	South Korea	LC270272	Unknow	2017
