# Supplementary material for: Detection of Persistent Viruses by High-Throughput Sequencing in Tomato and Pepper from Panama: Phylogenetic and Evolutionary Studies
Source: Plants (Basel). 2021 Oct 26;10(11):2295. doi: 10.3390/plants10112295 (PMC8620285; doi:10.3390/plants10112295)
Supplement: Supplementary file 1 [file plants-10-02295-s001.zip › Suplementary Table S3 (Galipienso et al., ).pdf]

**Isolate**	**Country**	**Accesion n.**	**Host**	**Variety**	**Collection year**
BPEV_Panama	Panama	MZ122790	*C. annumm*	Unknown	2018
BPEV_EL Ejido	Panama	-	*C. annumm*	Local Idiap149	2018
BPEV_Tierra Blanca 1	Panama	-	*C. annumm*	Local Idiap149	2018
BPEV_Tierra Blanca 2	Panama	-	*C. annumm*	Local Idiap149	2018
BPEV_N65	Slovakia	MN580384	*C. annumm*	Promotor	2017
BPEV_TW	India	KU923756	*C. annumm*	Taiwan2	2015
BPEV_DR	Dominican Republic	KX522567	*C. annumm*	Unknown	2013
BPEV_IS	Israel	JQ951943	*C. annumm*	Atir	2011
BPEV_Ontario	Canada	KT149366	*C. annumm*	Healy	2014
BPEV_Santa Fe	Colombia	KX977568	*C. annumm*	Unknown	2015
BPEV-YW	USA	JN019858	*C. annumm*	Yolo Wonder	2007
BPEV_Penol	Colombia	KX977569	*C. annumm*	Unknown	2015
BPEV_lj	China	KF709944	*C. annumm*	Unknown	2012
BPEV_Kyosuzu	Japan	AB597230	*C. annumm*	Kyosuzu	2011
BPEV_Antioquia May5	Colombia	MN073197	*C. annumm*	Unknown	2018
BPEV_MS1	Ecuador	MN175323	*C. annumm*	Unknown	2017
BPEV_XJ	China	MH182675	*C. annumm*	Luosijiao	2017
BPEV_LA-E	USA	MT013204	*C. frutescens*	Unknown	2017
