# Supplementary material for: Detection of Persistent Viruses by High-Throughput Sequencing in Tomato and Pepper from Panama: Phylogenetic and Evolutionary Studies
Source: Plants (Basel). 2021 Oct 26;10(11):2295. doi: 10.3390/plants10112295 (PMC8620285; doi:10.3390/plants10112295)
Supplement: Supplementary file 1 [file plants-10-02295-s001.zip › Supplementary Table S7 (Galipienso et al., ).pdf]

| Name | Sequence (5’-3’) | Genomic region |
|---|---|---|
| BPEV_F | GGT ACA AAT TTA GTG CAC ACC G | 11875-11897 (Polyprotein) |
| BPEV_R | TCT CGG TTC CAA TTT CGG TC | 12093-12073 (Polyprotein) |
| BPEV probe | 6FAM-TGG ATA AA TGA CGG CAC ATG TTG GGA-TAMRA | 11992-12019 (Polyproyein) |
| STV_F | TGC CTC CCC AGC TGT CA | 1189-1206 (CP/RdRp) |
| STV_R | TGC GTT GGG ATA GAG GAG TGA | 1236-1257 (CP/RdRp) |
| STV probe | 6FAM-CGC AAC AGA GGT AGA GGC AGA GGC C-TAMRA | 1209-1234 (CP/RdRp) |
